# Her Heart's Mistake

**Published:** 1889-05-18

**Authors:** E. D. Cumming

**Affiliations:** Author of "An Ordeal by Silence"


### Her Heart's Mistake.

By E. D. Cumming, Author of "An Ordeal by Silence."

(Continued from page 92.)

Chapter VI. The Hurdle Race.

"But, George, dear, hadn't we better wait until you can get a good long spell of leave? I want to have you entirely to myself for the first month or two after we're married."

"Well, Kitty, I had looked forward to calling you my wife before many weeks were over, but I quite recognise the justice of your reasoning. Are you going to make me wait until the cold weather?" asked Bairdsley, with a smile.

"It will come in November, George, and we are nearly half way through July now. Is it too long to wait?"

"Very much too long. How do I know you won't run away in the meantime?" he asked, laughingly.

"Well, George, if there is any talk about the regiment or papa being ordered away from Meerut, I'll relent, and we will marry at once. I couldn't consent to part with you now. You know that papa is anxious to retire and go home at the end of the year, and he makes a great point about keeping me with him until he goes. That's another reason I have for asking you to wait.

"Your reason does you credit, dear. It's like you to think of others. Perhaps it would be more convenient to wait, as I have to order heaps of things from home. Oh, its a fearful nuisance getting married," he sighed, with a movement which, to say the least of it, was contradictory.

This conversation took place in the drawing-room of the corner bungalow, after a nondescript meal, half lunch, half tea, with which the partakers were fortifying themselves for the Sky Meeting.

"Now, Kitty, I don't want to hurry you, but I've got to ride Red Hazard in the first race, and ought to be on my way," said George Bairdsley, looking at his watch. "The race starts at 4.45, and it's a quarter-past four now.

"Order the gharry, dear. It's too hot for the open carriage; and I'll drive up with you at once."

Bairdsley went out to the verandah and shouted instructions to the stable, where half the syces were busy "malishing" Baroness in preparation of her first public appearance on the turf. He had seen his antagonists, Monk and Volunteer, taking their morning breathers, and after giving Baroness a quiet gallop two days before, when the racecourse was deserted, had come to the satisfactory conclusion that, if temperately ridden, the mare would have a very fair chance of catching the judge's eye.

The bell, warning riders to weight out for the first race, was ringing when Kate and her escort drove up to the gate of the stand enclosure; and having taken her upstairs and found her a seat which commanded a good view and was well sheltered from the sun, Bairdsley ran down to the dressing-room to prepare for the race.

The grand stand looked very forlorn this afternoon. Other ladies had not the inducement that Kate had to come early, and the benches were occupied by a few Eurasians, members of that anomalous class which owes its being to Europeans and Asiatics, and is shunned by both. Kate knew none of them, for Meerut society held itself aloof from the half-caste population, so she turned to the scene outside, which was growing more animated every moment. Natives were flocking in hundreds to the ropes struggling good-humouredly for places whence they could see the course. Sowars of the Punjaub Cavalry were riding slowly up and down to keep the space between the ropes clear. Half-a-dozen native policemen were hunting the pariah which haunts the Indian turf as faithfully as the stray dog does the English racecourse. Round the wide level maidan, parched and sun-baked, the course itself ran like a green ribbon; for the galloping track of a large Indian station is as fondly nursed and tended through the hot weather as a tennis-maniac's lawn is at home. It had been well watered during the past few days, and only eyes which ached from continued contemplation of arid waste could properly appreciate the sight of young grass sprouting fresh and green, like an emerald stream on the dusty plain. Immediately below, in the stand enclosure, native officers and gentlemen congregated, conversing with civilians and other white men, with whom the great business of racing gave them for once a common interest. There was none of the rowdyism of an English course to be seen: everyone was orderly and well-behaved.

The first race was run, and placed to the credit of Red Hazard, whose rider had an easy task to pull it off. While preparations were going on in the weighing-room for the next event people began to arrive, and when the saddling-bell rang the stand was comparatively full.

"I knew I should find you here," said Mrs. Arkwright to Kate. "I should have come sooner myself, but two or three men came in to ask for a cup of tea—which is a polite fiction for a whisky-and-soda, as you may have learnt ere now. Who won the half-mile?"

"Red Hazard," answered Kate. "Your husband was third. I see he is going to ride in this race. Have you been betting on him?"

"I shall, if anyone offers to—one always feels it a duty to back one's husband; but Jack never wins by any chance. If I were to pay all the glove debts I have lost through my wifely loyalty, I should be ruined."

The bell for the horses to go out and face the starter rang at this moment, and Mrs. Arkwright stood up to watch them come out of the paddock.

"Are you going to have some gloves on this event, Mrs. Arkwright?" asked a man's voice behind her.

"I'm always glad to oblige, Captain Andrews. The usual thing, I suppose?"

The "usual thing" appeared to be for the lady to select half the field, and leave the remainder for the gentleman—a somewhat one-sided arrangement for the latter, who was, moreover, called upon to give disinterested advice on the merits of the various horses, to enable the lady to choose her representatives.

"I think I ought to win that," she observed, studying her race-card when the division had been made. "I've got both Prince and Formosa."

"As Prince can give any other animal in the race fourteen pounds and gallop round him, I think you ought to win, unless he tumbles down and all your others tumble over him."

Captain Andrews' appointment as Mrs. Arkwright's "bow-wow" cost him about fifty rupees in gloves every race meeting, but as he never complained no one else raised objections. The sad-eyed Tops, who had been crushed to the earth by the news of Miss Harrison's engagement, came to beg that she would have a bet with him on the same principle; and Kate, having insisted upon a fair allotment of horses, allowed him to pick out her share; an undertaking which he performed with extreme conscientiousness, but which, to his chagrin, resulted in his assigning the winner to himself.

"We must have another on the next race, Miss Harrison," he said, earnestly, "to give you your revenge."

Regard for the young officer's purse always led Kate to sternly refuse payment of her winnings, but Tops would not brook such treatment. Perhaps if the young lady had known the pleasure it gave him to send her gloves and receive her little note of thanks in return, she might have been less scrupulous.

"Very well, Mr. Wentworth," she replied; "but remember that if I win this time we shall be quits."

The third race was over, and George Bairdsley came to take her down to the paddock where Baroness was being walked round by her syce, who had much ado to keep the mare from breaking away from him altogether.

"She's a little excited with the row and the crowd," said Bairdsley; "when I get her over to the other side, at the starting-post, she will calm down. Will you wait here, while I change my jacket and weigh?"

Kate put up her sunshade and stood there, awaiting his return; she felt a little anxious about letting the mare run in her first jump-race when she was so much "above herself," as she appeared to be this afternoon.

"My dearest girl, it's all right," said Bairdsley, to whom she confided her fears when he came back, followed by a syce carrying weight-cloth, saddle, and bridle. "Believe me, she will go like a lamb when once I get her out of the paddock."

He stood by her directing the operation of saddling, and waited until all the others had obeyed the "Go out" bell.

"I'm not going to stand about in the sun any more," he said; "nearly ten minutes to wait at the post in that second race before Arkwright thought fit to turn up, and then five false starts in the last one is enough for me."

"I do hope it's safe, George," said Kate, as a soldier gave him a leg up. "I should never forgive myself if anything happened."

He reassured her, and she went upstairs to her place in the stand again, whence she watched him ride out with much the same feelings as those with which a lady of the middle ages might have seen her armoured champion prick forth to knightly combat in the lists. She felt a proud, loving sense of ownership as Bairdsley sent Baroness past the stand at a long swinging stride in the preliminary canter. He was wearing colours which all might see matched her dress, and her heart swelled with delight as she saw the mare's glossy chestnut coat flashing gold in the sun, and noted the perfect control her rider possessed over the excited Arab. Bairdsley pulled up at the corner to receive his solah topee from the syce who held it, and putting it on over his cap, which was no protection from the still powerful sun, trotted slowly over to the starting-post, where the field was assembling. Kate took up the race-glass with which he had furnished her, and watched the horses moving up towards the starter's upraised flag, telling off those she recognised as she did so. "Black and red; that's Major Arkwright on Volunteer. Chocolate, yellow sleeves, and cap; that's Mr. Pearson, on Forester. Blue, white cap; that is Major Wilton, on Monk. I like our colours best. Oh, they're off!"

The flag fell to a capital start, and the whole field of eight horses rose like one to the first hurdle, which stood within a short distance of the post. They were spreading out a little when they reached the second one, but the last horse was over it within twenty yards of the leader. Kate could see that Volunteer was in front, with Forester, Baroness, and Monk in close attendance.

"Pretty race," murmured Captain Andrews, as the field, still well together, came round the corner, leaving an immense column of dust in its wake. "I wouldn't like to ride a very backward race with all that dirt flying about. Impossible to see a jump through it. Ah, I thought so, someone is down! Can you see who it is, Miss Harrison, with your glass?"

"Pink," briefly answered Kate, who was for the time living in the race.

"The festive Tops," said Captain Andrews. "I see the horse isn't down. I bought Schoolmaster in two lotteries last night on receiving Wentworth's solemn assurance that he would tie himself on. Some fellows have no sense of honour at all."

"You couldn't expect Mr. Wentworth to tie himself on to a horse," said Mrs. Arkwright, who was apt to take all things seriously. "He might kill himself."

"Yes, he might of course," answered the speaker carelessly, "but Schoolmaster might have won if he hadn't tumbled off."

Mrs. Arkwright turned away, not quite sure whether her *attaché* was in earnest or not, and bent her eyes upon the race again.

The three leading horses, Forester, Baroness, and Volunteer, had negotiated the fourth hurdle, and were rounding the bend into the straight. Forester was showing the way, and half a length separated each of the three; but it was obvious that Bairdsley was only lying second in order to obtain Forester's lead over the hurdles. The dense dust concealed the other horses, but, as all interest was now centred in the first three, the fact was unimportant except to those unfortunates who had to make their way through the hot suffocating cloud. The fifth hurdle was safely passed, and the excitement began. Forester, slightly pressed by his rider, drew away for a couple of lengths, and Volunteer's jockey laid his horse alongside Baroness, whose pilot seemed quite satisfied to let his opponents do what they would.

"Bairdsley is riding that race like a workman," said Colonel Tenterdale as he saw the state of affairs. "If he gets the mare over the last hurdle, he'll make as pretty a finish of it as we've seen for many a long day. Halloa! What's Arkwright doing?"

Major Arkwright had taken his horse by the head, and was sending him up to the leader. Forester cleared the last hurdle in his stride, but Volunteer, thrown out by the injudicious hustling of his jockey, blundered and struck it heavily. Two bars were seen to fly out of it as Baroness rose to the leap, and in another second the mare was turning a somersault, while Bairdsley shot violently against a post, where he lay motionless.

"Tripped, by Jove!" exclaimed the Colonel, "and a nice race spoiled."

He ran out of the paddock, followed by a number of other men, and Kate, who had turned as white as death, prepared to go also.

"You mustn't go, my dear," said Mrs. Pringle, interposing. "Wait for a few minutes."

But Kate looked at her in dumb agony, and pressed her way out. He was hurt; he had not moved since he fell, and she must go to him.

An excited gesticulating crowd surrounded the group of Europeans who had come to Bairdsley's aid, and as Kate drew near she heard the rough voices of soldiers shouting to the natives to keep back. She took Colonel Tenterdale's arm, and he forced a passage through the throng until they came to the ring of white men, in whose midst Dr. Harrison and two other surgeons were examining the injured horseman. The confused sound of eager voices told Kate that he was at least alive. "Keep back and give him air!" "Hold up his head!" "Where's the brandy, give him another sip." "Never mind the buttons. Rip up his shirt, and let's get at the shoulder."

He was coming to himself when Kate appeared upon the scene, and all her self-control was required to keep calm. The blood was pouring down Bairdsley's face and shoulders from an ugly cut on the head, which was the least serious of his injuries.

"What is wrong?" whispered Colonel Tenterdale to Dr. Harrison.

"Not so much as we thought at first; concussion, dislocated shoulder, and badly sprained wrist. Quite enough for one day."

"How do you feel now, old fellow?" asked the Colonel, as Bairdsley looked round him in bewilderment, and tried to raise his arm to wipe the blood from his eyes.

"Trifle sick," gasped the sufferer. "Deuced lot of bone in-ground."

"We must put your shoulder in before you can move,' said Dr. Harrison. "The sooner it's done, the easier it will be for you. Have another nip of brandy first."

He took the stimulant, and the doctors laid him on his back on a horsecloth offered by a thoughtful syce, who was peering between the legs of the ring of soldiers.

"You're the strongest of us, Barker," said Dr. Harrison to a young army surgeon, who had been assisting him." "Kick off your boot and look sharp. Go away, Kate, we'll bring him along to the stand in two minutes."

"No; don't go," said Bairdsley. And Kate stayed.

Dr. Barker drew off his boot, sat down, and placed his stockinged foot under the patient's armpit, and then took his wrist in both his hands.

"Lucky it isn't this one that's damaged," he muttered to himself as he got into position. "Ready?" Bairdsley nodded, and clenched his teeth. The operator threw all his strength into a mighty pull. Dr. Harrison manipulated the shoulder, and in a few seconds, which the patient spent in kicking to relieve the torture he bore in silence, the dislocated joint was put in place.

"You're a tough subject," said Dr. Barker with an approving smile. "Painful job, isn't it?"

A bucket of water had been brought from the stand, and Kate knelt down at his side to wash the blood from his face, to which Bairdsley feebly objected.

"You'll make your dress in a fearful state," he objected; "don't do it."

"Be quiet," said Kate, who felt that she could only disguise her real feelings in severity. "They have sent for a dhooly, and you're coming home with us."

But Bairdsley insisted that he was all right, and in proof of his assertion rose to his feet. Colonel Tenterdale buttoned a coat round him, and took his arm to help him back to the dressing-room, while Kate placed herself at his side.

"I hope Baroness isn't hurt," said Bairdsley, as they entered the enclosure.

"Oh, no; she's all right," answered Kate.

"Have you looked at her knees?"

"Never mind about Baroness," answered the young lady, who had not looked at her favourite or even noticed her as she trotted into the paddock. "She is all right."

The dhooly—a covered stretcher slung on a pole—was brought from the hospital, and, to Bairdsley's disgust, the doctor ordered him into it.

"Why can't I drive home?"

"Because the road's too rough, and your shoulder would not be improved by the jolting. Come, lie down, and we'll follow you home."

"Take the sahib to my house," said Dr. Harrison to the dhooly-bearers. And calling Kate, who had stopped to see that the patient's pillow was high enough and do other small services, he summoned the carriage, and they drove rapidly away to get a room ready at the corner bungalow.

"How do you feel?" asked the Doctor, as the dhooly was laid on the verandah.

"Rather seedy; it's the smash on the head, I suppose."

"Of course. I'm afraid you'll be on the sick list for a day or two, but we'll look after you. Kate's a splendid nurse."

So George Bairdsley was comfortably installed as an inmate of the bungalow, and, in the prospective pleasure of nursing him, Kate almost forgot her remorse at Baroness's first race having caused the accident

(*To be continued.*)

